# The historical and current research progress on jujube–a superfruit for the future

**DOI:** 10.1038/s41438-020-00346-5

**Published:** 2020-08-01

**Authors:** Mengjun Liu, Jiurui Wang, Lili Wang, Ping Liu, Jin Zhao, Zhihui Zhao, Shengrui Yao, Florin Stănică, Zhiguo Liu, Lixin Wang, Changwei Ao, Li Dai, Xiansong Li, Xuan Zhao, Chunxiang Jia

**Affiliations:** 1 Research Center of Chinese Jujube, Hebei Agricultural University, Baoding, 071001 Hebei China; 2 College of Horticulture, Hebei Agricultural University, Baoding, 071001 Hebei China; 3 College of Forestry, Hebei Agricultural University, Baoding, 071001 Hebei China; 4 College of Life Science, Hebei Agricultural University, Baoding, 071001 Hebei China; 5 Department of Plant and Environmental Sciences, Sustainable Agriculture Science Center at Alcalde, New Mexico State University, 371 County Road 40, Alcalde, NM 87511 USA; 6 Faculty of Horticulture, University of Agronomic Sciences and Veterinary Medicine of Bucharest, 011464 Bucharest, Romania; 7 College of Food Science and Technology, Hebei Agricultural University, Baoding, 071001 Hebei China; 8 National Engineering Research Center for Agriculture in Northern Mountainous Areas, Hebei Agricultural University, Baoding, 071001 Hebei China; 9 Beijing Collaborative Innovation Center for Eco-Environmental Improvement with Forestry and Fruit Trees, Beijing, 100000 China; 10 Propaganda Department, Hebei Agricultural University, Baoding, 071001 Hebei China

**Keywords:** *Ziziphus jujuba* Mill, Fruit trees, Scientific research, History, Advances, Prospects, Plant sciences, Genetics

## Abstract

Jujube (*Ziziphus jujuba* Mill.), or Chinese date, is the most important species of Rhamnaceae, a large cosmopolitan family, and is one of the oldest cultivated fruit trees in the world. It originates from the middle and lower reaches of the Yellow River, the ‘mother river’ of the Chinese people. It is distributed in at least 48 countries on all continents except Antarctica and is becoming increasingly important, especially in arid and semiarid marginal lands. Based on a systematic analysis of the unique characteristics of jujube, we suggest that it deserves to be recognized as a superfruit. We summarized historical research achievements from the past 3000 years and reviewed recent research advances since 1949 in seven fields, including genome sequencing and application, germplasm resources and systematic taxonomy, breeding and genetics, cultivation theory and techniques, pest control, postharvest physiology and techniques, and nutrition and processing. Based on the challenges facing the jujube industry, we discuss eight research aspects to be focused on in the future.

## Introduction

Jujube (*Ziziphus jujuba* Mill.), also called Chinese date or Chinese jujube, is one of the oldest cultivated fruit trees in the world and is the most important species in the large cosmopolitan family Rhamnaceae in terms of its economic, ecological, and social importance. Its utilization and cultivation history can be traced back to the Neolithic age, 7000 years ago^1–5^. It has been spread all over China, with a cultivation area of ~2 million hectares and an annual production of over 8 million tons. More than 90% of jujube production is concentrated in six provinces, namely, Xinjiang, Hebei, Shandong, Shanxi, Shaanxi, and Henan. At present, it is one of the main cultivated fruit species, the foremost dried fruit in terms of production, and the main income source of ~20 million farmers in China^2,3^. Since it was introduced into neighboring countries such as Korea and Japan 2000 years ago, jujube has spread to at least 48 countries, commercial jujube cultivation has developed at different levels in China, South Korea, Iran, Israel, the United States, Italy, Australia, and other countires^2,3,6–8^. Jujube is becoming increasingly important in arid and semiarid marginal lands because of its outstanding endurance and adaptability to drought as well as barren and salty soil and deserves to be considered a superfruit for the future due to its distinct advantages. This paper will systematically summarize its special characteristics and comparative advantages, historical and recent research advances, and future research prospects to guide researchers and professionals working on jujubes.

## A superfruit for the future

A superfruit species for the future should simultaneously meet the diverse needs of growers, consumers, marketers, governments, and society. Generally, growers prefer fruit trees that crop early, reach high and stable yields quickly, and have light pest pressure, easy management, low cultivation costs, and high economic benefits. Consumers like fruits that are delicious and nutritious and that appeal to appearance and status. Marketers prefer fruits that can be easily transported from production areas and have long shelf lives and large markets. The government and society pay more attention to ecological friendliness, the efficient use of land resources and the social and economic advantages for rural farmers in marginal regions. Jujube clearly deserves to be considered a superfruit for the future in terms of its following basic characteristics.

**Fig. 1:**
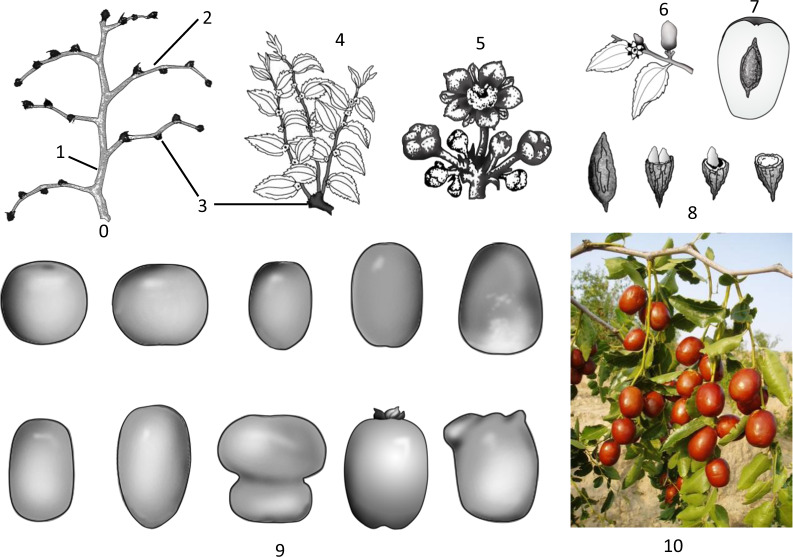
The morphology of different organs of Chinese jujube. 0—perennial shoot system in the dormant season, 1—primary shoot, 2—secondary shoot, 3—mother-bearing shoot, 4—fruit-bearing shoot, 5—inflorescence, 6—young fruit, 7—longitudinal section of mature fruit, 8—stone and kernel, 9—the diverse shapes of jujube fruits, 10—photograph of fruiting jujube

First, jujubes can satisfactorily meet the various needs of growers. It can blossom and even bear fruit in the same year as planting or grafting and can achieve high yields 3–5 years after the establishment of an orchard under a high-density planting system. Benefiting from late bud sprouting (~20 days later than peach), late flowering (June in North China) and a 2-month flowering period, jujube avoids late frosts and biennial bearing. It only takes 10 days to differentiate flowers whenever there is new shoot growth^1^. Jujube never needs flower-promoting treatments. It has a very low chilling requirement and is suitable for protected cultivation. It only needs light pruning because of its unique self-pruning habits: fruit-bearing shoots fall off in the fall, mother-bearing shoots extend only ~1 mm a year, secondary shoots die back year by year, and only the tops of the primary shoots can extend (Fig. 1). Jujube is highly tolerant of drought, infertility and salinity, and has low demands for water and fertilizers^4^ (Table 1). Consequently, the costs of cultivation and management for jujube are significantly lower than those for other common fruit trees.

**Table 1:** Jujube growing conditions in China

Climatic condition	Soil condition		
Items	Value	Items	Value
Annual average temperature (°C)	5.5–22	Depth (cm)	≥30
Average temperature in the flowering season (°C)	≥22–24	pH	4.5–8.4
Minimum temperature (°C)	≥−38.2 °C	NaCl (%)	≤0.15
Frost-free days (d)	≥120	Na_2_CO3 (%)	≤0.3
Annual rainfall (mm)	20–2000	Na_2_SO4 (%)	≤0.5
Annual sunshine (h)	≥1100		

Second, jujube fruits can satisfactorily meet the various needs of consumers. Its fruit is smooth and bright, like a large red pearl. The flesh is as sweet as honey and as crisp as an apple or pear. The fruit is particularly rich in nutrients, and its contents of sugar, vitamin C and B, cyclic nucleotide, proline, triterpenic acid, potassium, iron, and zinc are the highest among many fruits^9,10^ (Table 2). The contents of sugar, vitamin C, and cyclic adenosine monophosphate (cAMP) are around 2, 100, and 1000 times those of apple, respectively. The fruit is also a rich source of polysaccharides, triterpenic acids, flavonoids, alkaloids, polyphenols, and pigments^3,4,11^. It is a medicine/food homolog and a famous commonly used traditional Chinese medicine that is used in ~50% of Chinese herbal medicine prescriptions. Jujube fruit also has very positive meanings in Chinese culture, such as a sweet life, a flourishing business, fertility, harmony, and happiness.

**Table 2:** The nutrient profile of jujube fruit and other fresh fruits and nuts

Nutritional component	Fresh jujube	Dried jujube	Sour jujube	Apple	Orange	Walnut	Chestnut
Carbohydrates (%)	23–32	63.0–76.3	74.8	11.5–13.2	9.4–12.8	5.4–10.9	39.9
Vitamin C (mg/100 g)	200–800	12–29	–	1–6	34	3	60
cAMP (μg/g)	–	40-400	–	0.01			
Water (%)	73.4	14–27.8	16.8	84.6–87.0	85.4–89.2	3.0–4.0	54.0
Protein (%)	1.2	2.9–6.3	4.5	0.2–0.4	0.6–0.9	15.4–19.6	4.0
Fat (%)	0.2	0.3–2.3	1.0	0.2–0.5	0.1–0.3	63.0–69.0	1.1
Coarse cellulose (%)	1.6	1.8–3.1	0.2	0.6–1.2	0.3–0.4	1.1–6.6	1.0
Ca (mg/100 g)	14	20–63	270	5–22	20–56	43–119	15
P (mg/100 g)	23	55–75	59	3–9	8–20	329–386	77
Fe (mg/100 g)	0.5	1.6–3.1	3.8	0.2–1.0	0.2–2.0	2.9–3.9	1.5
Carotene (mg/100 g)	0.01	0.01	–	0.05–0.4	0.55	0.16–0.17	0.02
Aneurine (mg/100 g)	0.06	0.06	–	0.01–0.03	0.08	0.30–0.32	0.07
Lactoflavin (mg/100 g)	0.04	0.3	–	0.01–0.03	0.03	0.11–0.16	0.05
Niacin (mg/100 g)	0.6	1.2	–	0.1	0.3	1.0–1.7	1.0

Data from the authors’ research and the publication ‘Food Component List’ compiled by the China Sanitation Research Institute^9,10^.

Third, jujube fruit can satisfactorily meet the various needs of marketers. The fruit can be stored and transported easily. Dried jujube (the main form) can be stored for more than 1 year at room temperature with proper insect control and for 2–3 years at 4 °C. Fresh jujube fruit can be kept fresh for 2–4 months under controlled atmosphere storage or controlled freezing-point storage. It has multiple uses as a fresh or dried fruit, processing material, traditional Chinese medicine, celebratory food, health product, and a representative of native Chinese products. It has great potential in international markets, as currently, its export proportion is <0.5% in China. It can be seen that jujube fruit can meet the marketer’s demand for profits and risk avoidance.

Fourth, jujube can satisfactorily meet the concerns and needs of the government and the public. Jujube trees are highly adaptable, with outstanding tolerance to drought; low-fertility, saline, and alkali soils; high temperatures and late frosts. It can grow and bear well where normal grain, cotton, oil crops, vegetables, and fruit trees cannot survive. Therefore, the development of jujube can make efficient use of marginal lands, improve the environment, and provide farmers in rural regions with an agricultural industry that is easy to manage and provides high revenues.

Jujube can satisfactorily meet the diverse needs of growers, consumers, marketers, government, and society. Its fruit has the five basic elements of a superfruit, i.e., delicious taste, attractive appearance, nutrient density, safe consumption, and strong cultural meaning. It is expected to become one of the most promising fruit trees in the arid and semiarid regions of the world. Therefore, jujubes will attract increasing attention worldwide. Jujube fruit can become a part of international markets and a future popular superfruit.

## Research progress in the past 3000 years

### Historical achievements and contributions

**Fig. 2:**
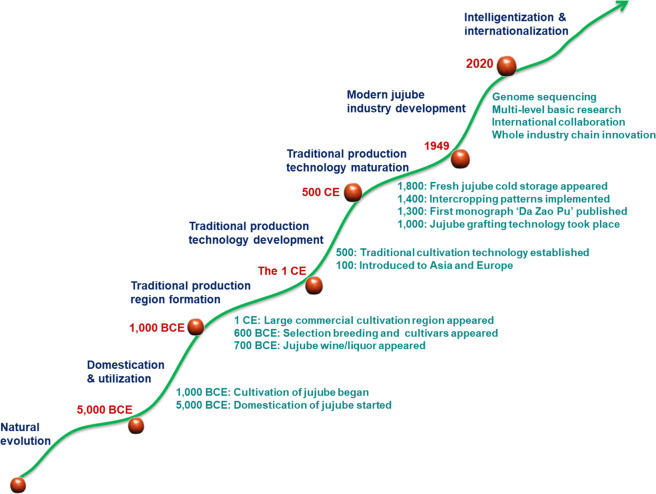
The trend of jujube industrial development and research

Around 79.9 million years ago, jujube diverged from the families Moraceae and Cannabaceae^12^. Twenty-four million years ago, jujube trees appeared in Northern China^10^. At 7240 years ago, the domestication of jujube trees started^1^. The oldest collection of poems in China, called ‘The Book of Songs’, contains the sentence ‘Jujube fruit picked in August and rice harvested in October’, indicating that jujube was domestically cultivated as early ago as 3000 years ago. In addition, 11 jujube cultivars were recorded in ‘Erya’, a book written 2600 years ago. Moreover, 2000 years ago, jujube had spread throughout China, large-scale jujube commercial production areas had developed, and jujube had become one of the five most important fruit trees in China, namely, peach, apricot, plum, chestnut, and jujube. In addition, 2000 years ago, jujube was introduced into Japan and Korea, to Central Asia and Europe via the ancient Silk Road, and gradually into at least 47 countries (Table 3) on all continents except Antarctica (45 °N–35 °S)^1–3,5^. The book ‘Qi Min Yao Shu’ of the Northern Wei Dynasty that was written 1500 years ago recorded the techniques of cultivar selection, planting, flower thinning, girdling, and jujube paste processing. Jujube grafting took place 1000 years ago, and the intercropping of jujube with cereal crops had become popular by about 600 years ago. Thus, the level of jujube production in China has long been at the forefront of fruit tree production in history, and jujube production has made an important contribution to the development of fruit tree science and technology (Fig. 2).

**Table 3:** The worldwide distribution of jujube

Continent	Country
Asia	Afghanistan, Armenia, Azerbaijan, Bengal, Burma, China, India, Iraq, Iran, Israel, Japan, Kuwait, Kyrgyzstan, Lebanon, Malaysia, Mongolia, North Korea, Pakistan, Palestine, South Korea, Syria, Thailand, Turkey, Turkmenistan, Uzbekistan
Europe	Bulgaria, Cyprus, Czech Republic, England, France, Germany, Greece, Italy, Macedonia, Moldova, Portugal, Romania, Russia, Slovenia, Spain, Ukraine
Africa	Egypt, Tanzania, Tunisia
North America	Canada, USA
Oceania	Australia, New Zealand

## Research history

China is the country with the longest history of jujube cultivation. To date, more than 95% of jujube production is still concentrated in China, and scientific research on jujube is also mainly carried out in China. Here, we took China as an example to briefly introduce the historical evolution and major achievements in jujube production and scientific research over the past 3000 years. Jujube research can be roughly divided into five periods as follows^10^.

*The domestication period* (late Neolithic to the Shang Dynasty—3000 YA). According to leaf fossils, jujube appeared in northern China 24 million years ago. Neolithic charred fruits and pits of jujube unearthed in Peiligang, Mixian County, Henan Province, China, indicate that Chinese people had begun to pick and use jujubes and domesticate jujube trees at least 7240 years ago. Moreover, at the end of the Shang Dynasty, the description of ‘Jujube picked in August’ in the Book of Songs indicated that jujube orchards had appeared by at least 3000 YA.

*The traditional production region formation period* (Zhou Dynasty–Han Dynasty, or 1000 BCE–1 CE). Eleven jujube cultivars were recorded in ‘Erya’, written 2600 YA. By the Han Dynasty (~2000 YA), jujube cultivation had spread throughout North and South China into large commercial cultivation regions and had become the main source of national taxes, with special officials regulating jujube production. During this period, jujube began to be introduced to neighboring countries in Asia, such as Japan and South Korea, and began to spread to Central Asia and Europe through the ancient Silk Road. Gaius Plinius Secundus (AD 23–79), better known as Pliny the Elder, mentioned in his ‘Historia Naturalis’ that a counselor of the Roman Emperor Octavian Augustus introduced the Chinese date (jujube) for the first time from Syria to Italy; from there it was distributed to other Mediterranean countries and around the Black Sea basin^13,14^.

*The traditional production technology development period* (end of Han Dynasty–Northern Wei Dynasty, or 1 CE–500 CE). Twenty-one jujube cultivars were recorded in the book ‘Guangzhi’ written by Guo Yigong in the Jin Dynasty, 1700 YA. Approximately 1500 YA (the Northern Wei Dynasty), Jia Simiao wrote a famous agricultural book called ‘Qi Min Yao Shu’ that not only recorded 45 jujube cultivars but also described cultivar selection breeding, orchard site selection, planting times and methods, flower thinning, girdling, harvesting, and jujube paste processing. This evidence showed that the traditional jujube cultivation technology system had been established at 1500 YA, and many of these processes have been used continually until the present.

*The traditional production technology maturation period* (Northern Wei Dynasty–the founding of the People’s Republic of China, or 500 CE–1949 CE). From 500 CE to 1949, the cultivation of jujube mostly followed traditional methods. The central and local governments encouraged jujube growing via legislation to increase farmer incomes and improve the environment. The major advances in this period included grafting techniques (1000 YA), intercropping (600 YA), fresh fruit storage in ice rooms (200 YA), shifting the girdling time from winter to the flowering season, and processing candied jujubes, jujube wine, and jujube vinegar. In this period, the first jujube research monograph ‘Da Zao Pu’, written by Liu Guan (Yuan dynasty, 1300), which recorded 71 jujube cultivars, was published.

*The modern jujube industry development period* (1949–present). The beginning of this period was marked by the founding of the People’s Republic of China in 1949. This period has lasted for over 70 years. In this period, the modern jujube industry system was initially established by the application of modern scientific technology. The development of the jujube industry and jujube research promoted each other during this period, and support from science and technology to the jujube industry has continuously increased. Based on the main contents of jujube research, the landmark achievements and the supporting role for jujube industry development, this period can be further subdivided into three stages^3^—the period of recovery after World War II (1949–1978), the period of accelerating development (1979–1999), and the period of comprehensive development (2000–2019). In this latest period, research achievements have been very fruitful. This period has not yet ended, and there is still a long way to go before establishing a modern technology system for jujube.

## Research achievements since 1949

During the past 70 years, jujube research has greatly advanced. A historic leap has been achieved from the initial stage of focusing on summarizing production experiences to a new era of innovative research throughout the whole industry chain covering breeding, cultivation, pest management, fruit storage, transportation, and processing; from morphological research to comprehensive research at the levels of the plant, organ, tissue, cell, molecule, and gene; and from the research of a few individuals to national and international research collaborations.

### Genome sequencing and its application

Genome sequencing is the golden key to unlocking the genetic code of life. Genome sequencing-based multiomics analysis combining transcriptome, proteome, metabolome, and phenotypic data has taken horticultural research to a new level.

#### De novo genome sequencing and pseudochromosome construction

In 2014, de novo genome sequencing of jujube was accomplished (the first in the Rhamnaceae family)^12^. A strategy combining WGS sequencing, BAC-to-BAC and WGS-PCR-free library was employed to address the impact of the high complexity of the jujube genome, which has a high heterozygosity of 1.90%, a low GC content of 33.41% and a high density of simple-sequence repeats (SSRs) (378.1 per Mb). The assembly (437.6 Mb) covered 98.6% of the estimated jujube genome (444 Mb), and 32,808 protein-coding genes were predicted (http://jujube.genomics.cn/page/species/index.jsp). Later, another jujube genome and the chloroplast genome sequencing of four *Ziziphus* species were published^15,16^.

Using an interspecific population between *Z. jujuba* and *Z. acidojujuba*, a high-density molecular (SNP) genetic map was constructed by restriction site-associated DNA sequencing^17^. The joint map across the 12 linkage groups (the same as the basic chromosome number in jujube) spanned 1020.22 cM, with a mean marker distance of 0.32 cM. Combining these genetic linkage groups with the assembled genome sequences, pseudomolecules for each of the 12 chromosomes of jujube were constructed. A total of 23,996 genes (73% of the total annotated genes) were allocated on the 12 pseudochromosomes. Self-alignment of the jujube genome sequences based on the 23,996 gene models identified 943 paralogous gene groups, indicating that the jujube genome may have undergone frequent intrachromosomal fusions and segment duplication during its evolutionary history. A gene block located in the region from 9.20 to 14.68 Mb of pseudochromosome 1 is highly conserved and contains many genes related to sugar metabolism and stress tolerance. An evolutionary divergence analysis of jujube, pear and *Prunus mume* found that the 4DTv (fourfold synonymous third-codon transversion) rate of jujube peaked at only 0.50, suggesting that no recent whole-genome duplication had occurred in jujube^12^.

*Genome-wide screening of SSR markers and reference genes for RT-qPCR analysis*. The density of SSRs in the jujube genome reaches 378 SSRs per Mb, which is approximately two times the density in peach and apple^12^. Using the genome data, 511 pairs of SSR primers showing high polymorphism were screened and applied for the identification of large-scale hybrid progeny and a genetic diversity analysis^18–20^. A total of 963 jujube germplasms were analyzed by SSRs to construct the core collection and germplasm resource management database^21^.

Specific reference genes for RT-qPCR of jujube were selected under a variety of conditions and sourced from different tissues/organs, fruit development stages, and biotic/abiotic stresses, providing more choices for further gene expression analysis and functional studies in jujube^22–26^.

*Multiomics-based analysis for the molecular formation mechanisms of some important traits*. Combining comparative genome, transcriptome, and metabolome data, the molecular mechanism underlying the high contents of ascorbic acid (AsA) and sugar in jujube fruit (~100 and 2 times those of apple, respectively) were revealed. The l-galactose pathway is the major route for jujube AsA biosynthesis, and the genes encoding the key enzymes involved in the biosynthesis pathway show high-level expression during fruit development^12^. Meanwhile, the expansion of the *MDHAR* gene family contributes to AsA regeneration. Further studies indicated that *GLDH* and *MDHAR* are the crucial genes in jujube AsA synthesis and recycling, respectively^27^. The high level of sugar accumulation in jujube fruit is due to the expansion and high-level expression of genes involved in sugar metabolism and transport. In addition, the distinct trait of the ‘bearing shoot falling in winter’ is related to the ethylene and abscisic acid (ABA) pathways^12^.

The genome sequencing of a drying jujube cultivar ‘Junzao’ and the resequencing of some cultivated and wild jujubes identified the selective sweep regions involved in acid and sugar metabolism and provided insights into an important domestication pattern in fruit taste^15^. Studies have also proven that sugar transport plays a significant role in sugar accumulation^15,28^ and jujube fruits have characteristics of nonclimacteric fruits^15,29^. The ethylene and ABA pathways are involved in regulating jujube fruit ripening^30,31^.

Some gene families involved in phytoplasma and cold stress and flower and fruit development were identified and analyzed at the genome level^22–24,32–34^. A series of gene families and metabolisms responsive to phytoplasma infection were studied, showing that photosynthetic, carbohydrate, and energy metabolism play crucial roles during phytoplasma infection^33^ and that the MAPK-WRKY pathway is responsive to phytoplasma stress^23,24,32^.

### Germplasm resources and systematic classification

#### Construction of a highly representative germplasm repository

As one of the longest-cultivated fruit trees in the world, jujube has abundant germplasm resources after undergoing long periods of natural evolution and artificial selection. From the 1950s to 1980s, the first nationwide jujube germplasm investigation was carried out; a total of 700 jujube cultivars and 30 wild sour jujube genotypes were determined through synonym and homonym identification and recorded in the book *China Fruit Tree Records-Jujube*^1^.

Based on the nationwide germplasm investigation, the National Chinese Jujube Repository was constructed in Taigu, Shanxi Province, by the Ministry of Agriculture and Rural Affairs of the People’s Republic of China. To date, a total of ~930 jujube genotypes have been preserved, accounting for at least 90% of the total jujube genotypes in the world. Another germplasm repository funded by the State Forestry and Grassland Bureau of China was constructed in Cangxian, Hebei Province, where ~640 germplasm accessions, including some excellent variants of the local leading cultivars, such as ‘Jinsixiaozao’, ‘Wuhezao’, and ‘Dongzao’, are maintained. In addition, Hebei Agricultural University collected and preserved ~200 accessions of sour jujube germplasm and some other species of the *Ziziphus* genus, such as *Z. mauritiana* Lam., *Z. spina-christi* Willd, and *Z. nummularia* Burm. f.

#### Evaluation of elite germplasms with unique features

To date, ~700 jujube cultivars and 100 sour jujube genotypes have been evaluated. The evaluations have covered morphological, agronomical, cytological, palynological, nutritional, and reproductive biological traits, as well as biotic resistance and abiotic tolerance^2,35–37^. A number of excellent accessions have been identified, including triploid genotypes such as ‘Zanhuangdazao’^38^, ‘Pingguozao’^39^, ‘Jinglinyihaozao’, ‘Shanxitedage’, ‘Hengshuibianzhizao’, ‘Zhenhuluzao’^40^; the mixoploid genotype ‘Dongzao 2’^41^; the male-sterile germplasms ‘JMS1’ and ‘JMS2’^42^; the self-fruitless and self-sterile germplasms ‘Huizao’, ‘Jinsixiaozao 39’, ‘Yuanlingzao’, and ‘Xiangzao’^43^; the tortuous-branch ‘Dongzao’^44^; the seedless germplasm ‘Wuhexiaozao’; genotypes that are highly resistant to witches’ broom disease such as ‘Xingguang’^45,46^; and genotypes that are rich in functional nutrients^47–54^.

#### Establishment of the germplasm database and platform

Three representative books on jujube germplasm have been published. The first is *China Fruit Tree Records-Jujube*, mentioned above. The second is *Germplasm Resources of Chinese Jujube*^10^, which recorded 1033 accessions of jujube (*Z. jujuba* Mill.), sour jujube (*Z. acidojujuba* Liu et Cheng) and Indian jujube (*Z. mauritiana* L.). The third is *The Illustrated Germplasm Resources of Jujube*^55^, in which pictures and research data for 250 cultivars (57 table jujubes, 80 for dehydration, 82 for both dehydration and fresh eating, 17 for processing, and 14 ornamental varieties) were obtained from the National Chinese Jujube Repository (Taigu, Shanxi). In addition, other books such as *Descriptors and Data Standards for Jujube Germplasm*^56^, *Test Guidelines for Distinctness, Uniformity and Stability—jujube*^57^, *Technical Regulators for the Identification of Jujube Cultivars-SSR Marker Method* provide references and technical standards for character selection, data collection, testing, and identification in jujube germplasm studies.

The established germplasm platforms include the following:

(1) Internet Information System for Perennial and Asexual Crop Germplasm Resources (http://www.ziyuanpu.net.cn/), which includes the data observed for many years at the National Jujube Repository in Taigu, Shanxi, China;

(2) Chinese Crop Germplasm Resources Information System—jujube (http://www.cgris.net/query/croplist.php), where germplasm checking and analysis can be performed; and

(3) Internet Information System for Jujube Germplasms (http://www.ziziphus.net/zzzy), which provided information observed from local areas about 700 germplasm accessions recorded in *China Fruit Tree Records—Jujube*.

In addition, the International Cultivar Registration Center for the *Ziziphus* genus was established in 2014 at the Research Center of Chinese Jujube, Hebei Agricultural University under the authorization of the International Society of Horticultural Sciences.

#### Clarification of the ancestor and the original cultivation center

In the book ‘Qi Min Yao Shu’, published 1500 YA, it is clearly recorded that ancient Chinese people used to select the trees with the best tasting fruits of wild sour jujube (*Z acidojujuba* Cheng et Liu—*Z. jujuba* Mill. var. *spinosa* Hu) and cultivate them^1–4^. Qu et al. proposed that jujube evolved from wild sour jujube^1^ based on a systematic study of ancient books, ecological distributions, karyotyping, palynology, and isoenzymes as well as of the transitional types between jujube and sour jujube^1^. A further cluster analysis of isoenzymes, palynology, and DNA showed that some jujube cultivars and sour jujube genotypes were usually clustered together instead of being totally separated^58–60^, indicating that there are probably several evolutionary pathways from sour jujube to jujube. This result was later confirmed by SSR and cpSSR analysis^61,62^.

As to the original cultivation center of jujube, outside of China, Iran, and Japan had been regarded as candidates by some scholars outside China. However, the earliest records and evidence from Iran and Japan could only be traced back to 2000 YA, when Sino–Japan exchanges became popular and Zhang Qian was sent on a diplomatic mission to west Asia and European countries during the Han Dynasty. According to the author’s investigation in Iran, all the jujube trees that were hundreds of years old were located at key sites on the ancient Silk Road, and Iranian scholars report that the jujube was introduced from China. In the *Book of Songs*, published 3000 YA, it is clearly mentioned that jujubes were already widely cultivated in China. Additionally, according to the unearthed carbonized fruits, jujube was cultivated and utilized in China 7000 years ago. It was reported that jujube originated from the Yellow River valley between Shaanxi and Shanxi provinces, China, after studying ancient documents, fossils and modern distributions as well as the transitional genotypes of jujube and sour jujube^1,5^.

#### Establishment of the taxonomic system for the Zizhiphus genus, including jujube

Based on the classical taxonomic system, jujube is listed in the Rhamnaceae family, Rhamnales order^63^. However, the Rhamnaceae family was moved to the Rosales order in the APG III Angiosperm Phylogeny Group Classification based on chloroplast DNA sequencing (Angiosperm Phylogeny Group III, 2009), which was further confirmed by phylogenic studies on the genome-sequenced plant species using 390 shared genes^64^.

The classification system for the *Ziziphus* genus was proposed based on field investigations, textual research on specimens and historical documents^65^. The genus *Ziziphus* was grouped into two sections based on geographical distribution, bearing shoot persistence, and leaf hairiness. They are Section *Ziziphus* Cheng et Liu and Section *Perdurans* Cheng et Liu. The latter was further divided into Ser. *Cymosiflora* Cheng et Liu and Ser. *Thyrsiflora* Cheng et Liu mainly according to the inflorescence type. Most species belong to Sect. *Perdurans*; only jujube and sour jujube originating from China and *Z. lotus* L., native to the Mediterranean, belong to Section *Ziziphus* due to their deciduous bearing shoots.

Regarding the taxonomic relationship between jujube and sour jujube (*Z acidojujuba* Cheng et Liu—*Z. spinosa* Hu), four viewpoints have been reported^66^, i.e., that they are the same species, that jujube is a variety of sour jujube, that sour jujube is a variety of jujube, or that they are two different species. Regarding the obvious differences in distribution, morphology, usage, and historical knowledge of jujube and sour jujube in China, Liu et al. proposed that they could be treated as two different species, *Z. jujuba* Mill. and *Z. acidojujuba* Liu et Cheng^67,68^. However, jujubes and sour jujubes are very closely related, with cross-compatibility and transitional types between them.

The subdivisions of *Z. jujuba* Mill. and *Z. acidojujuba* Liu et Cheng were also proposed to consider them as two independent species^67^. Under *Z. jujuba* Mill., five forms have been reported, i.e., f. *tortuosa* Cheng et Liu (*Z. jujuba* Mill. var. *tortuosa* Hort., *Z. jujuba* Mill. cv. Tortuosa), f. *lageniformis* (Nakai) Kitag. (*Z. sativa* Gatern. var. *lageniformis* Nakai, *Z. jujuba* Mill. var. *lageniformis* Hort.), f. *carnosicalycis* (Wang) Cheng et Liu (*Z. jujuba* Mill. var. *carnosicalycis* Wang), f. *allochroa* Cheng et Liu, f. *heteroformis* Cheng et Liu (*Z. jujuba* Mill. cv. *heteroformis* Hort., *Z. jujuba* Mill. var. *quinequeflora* Hort.), and f. *apyrena* Cheng et Liu (*Z. jujuba* Mill. var. *anucleatus* Y. G. Chen). Within *Z. acidojujuba* Liu et Cheng, three forms were confirmed, namely, f. *granulata* Cheng et Liu, f. *trachysperma* Cheng et Liu, and f. *infecunda* Cheng et Liu.

There appear to be at least 2, 16, and 6 scientific names for the jujube genus, the jujube and the sour jujube, respectively, due to different classification viewpoints and poor academic exchange in the past. Based on a systematic study of the historical taxonomic literature, Liu et al. affirmed *Ziziphus* (rather than *Zizyphus*), *Z. jujuba* Mill. and *Ziziphus acidojujuba* Cheng et Liu as the proper scientific names for the jujube genus, the jujube and the sour jujube, respectively^10,65–69^.

### Breeding and character inheritance

Jujube breeding has a long history. However, until the end of the 20th century, the breeding techniques for Chinese jujube had been mainly focused on selection from seedlings, bud mutants, and local germplasm, even though some new techniques had been incorporated into selection breeding, such as marker-assisted identification and standardized techniques. After entering the 21st century, great progress has been made in polyploidy and cross-breeding in jujube. Genetic engineering has also made some advances, but these applications are not yet fully developed.

#### Upgrading the breeding objectives

Breeding objectives should take into consideration the characteristics of jujube trees, the demands of all related parties, the breeding trends in fruit trees and breeding practices in jujube^70^. The overall objective should be to meet the various needs of farmers, processers, marketers and consumers. The specific objectives should include outstanding resistance to biotic and abiotic stresses, dwarfing, low branching ability, thornlessness, early bearing, high and stable yields, high quality, stonelessness, high nutrient levels, various ripening times, ease of transport and storage, and multiple-use cultivars. The major requirements to meet farmers’ needs are reducing inputs, increasing output and accelerating economic returns. Good quality, stonelessness, high nutrient levels, and varied ripening times can satisfy consumers, whose demands have become increasingly critical and diversified. Objectives such as tolerance to transportation and long storage life, varied ripening times, and multiple-use cultivars are marketers’ preferences. A revolutionary novel cultivar could simultaneously satisfy the diverse demands of farmers, processers, marketers, and consumers.

#### Creating a mixoploid-free polyploid induction system

Given the limitations of traditional selection breeding for obtaining breakthrough cultivars and the extreme difficulty of cross-breeding in jujube, polyploid breeding seems to be a promising prospect. Consumers usually prefer large fruits, but increasing fruit size by applying more fertilizer and plant regulators may result in poor fruit quality. As a result, polyploidy induction has become an ideal breeding approach for obtaining new cultivars with high-quality, large fruits.

**Fig. 3:**
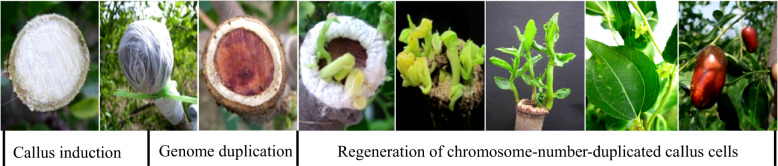
The process of in vivio induction of homogeneous polyploids via calluses in jujube

Four generations of polyploid induction techniques using colchicine as the main mutagen have been developed for jujube, i.e., in vivo apical bud induction^71^, in vitro apical or lateral bud induction^72^, in vitro callus/embryo induction^73^ and in vivo callus induction^74^. The fourth-generation technique is to induce polyploidy by treating in vivo calluses induced on branch cuts with colchicine, which could eliminate the severe mixoploidy formation from traditional polyploid breeding and directly produce pure polyploid shoots (Fig. 3). The pure polyploid shoot could form flowers and even set fruits for further evaluation in the same year as the polyploid induction. Consequently, the pure polyploid creation and evaluation period can be shortened by 3–5 years. Until now, a total of 25 triploid, tetraploid, and octoploid strains have been created, among which three excellent tetraploid strains have been released as new cultivars by the Hebei Forestry Cultivar Examination and Approval Committee^75–77^. The novel field technique for homogeneous polyploidy induction has been successfully applied in sour jujube^78^ and in elm (data not shown).

Auto-tetraploids differ greatly from their diploid counterparts in their morphology, cytology, and nutrient content^79^. Comparing the tetraploid jujube cultivar ‘Riguang’ and its diploid counterpart ‘Dongzao’, ‘Riguang’ has wider and darker green leaves, higher chlorophyll content, a higher photosynthesis rate, larger stomata, larger pollen and flowers, wider and larger fruits, and an earlier maturation time but is also less vigorous and less cold hardy^79^. The contents of vitamin C, cAMP, soluble sugars, titratable acids, sucrose, glucose, and fructose were significantly higher in tetraploid ‘Riguang’ fruits than in diploid ‘Dongzao’ fruits^79^. It was discovered that an autotetraploid sour jujube had higher tolerance to salinity than the diploid, and its preliminary molecular mechanism was illustrated^80,81^. In addition, triploids of ‘Jinsixiaozao’, ‘Yuanlingzao’, and ‘Changhongzao’ have been created by endosperm culture, but no new cultivar has been released^82^. Anther/pollen culture has also been practiced in jujube, and some plants have been generated from pollen^73,83^.

#### Establishing emasculation-free cross-breeding technology

Cross-breeding, the most powerful breeding method for fruit trees, has not been successfully utilized in jujube. This is a result of several key obstacles, including the extreme difficulty of emasculation of small flowers (~5 mm in diameter), the low fruit-setting rate (only ~1%) and the high embryo abortion rate. The rate of obtaining hybrids in jujube is usually <0.01% by the traditional crossing approach. Since no new gene fusions or multiple trait combinations are available in autopolyploidization and it is difficult to make large breakthroughs via selection breeding, there is no substitute for cross-breeding, and its advantages are also incomparable.

In the last 10 years, a high-efficiency hybrid breeding technology system combining male-sterile germplasm, embryo rescue, net control hybridization, and molecular identification was established. Hybrid plant production was increased by 100 times using the new system, and a large number of hybrid progeny were obtained from 19 cross combinations, of which a number of superior lines were selected^70^.

Three emasculation-free methods have been developed based on the discovery of two typical male-sterile germplasms and a group of self-fruitless/self-sterile germplasms that can replace male sterility^42,43^, which effectively overcame the key obstacle to artificial emasculation in jujube. Method 1: Hybrids are produced by using a male-sterile variety as the female parent and a variety with high pollen viability and compatibility as the male parent^20,84^. Method 2: A self-fruitless or self-sterile variety is chosen as the female parent, and a variety with high pollen viability and compatibility is chosen as the male parent. In the two cases mentioned above, all the offspring from self-fruitless or self-sterile parents can be directly regarded as authentic hybrids. Method 3: is a universal technology free of emasculation, i.e., covering the parents with nets to keep away unexpected pollen donors, pollinating by bees inside the nets, and identifying the hybrids with molecular markers^84^. This method can produce cross and reciprocal-cross hybrids at the same time^85^.

The problem of hybrids not being obtained due to heavy early embryo abortion was solved by embryo rescue based on the understanding of the mechanism of embryo abortion and the factors affecting very young embryo culture. These factors included the culture media, inoculation methods, removal or maintenance of the seed coat, combination of growth regulators, and concentrations of lactalbumin hydrolysate, activated carbon, and sucrose^86–88^. The seedling rate of young embryos <30 days after flowering (before the abortion peak) was increased from 3.7 to 40%^89^. The key techniques included peeling off the seed coat, culturing young embryos together with their endosperm and reverse inoculating young embryos with the chalazal end pointed down on the medium.

In recent years, the interploidy hybridization of jujube and the interspecific hybridization between jujube and sour jujube were successfully carried out^20,84,85^. With the breakthroughs in cross-breeding technology, the acquisition of a progeny population and the establishment of genetic maps, genetic research into important traits was also carried out.

Several high-density genetic linkage maps have been constructed based on segregation populations of ‘JMS2’ × ‘Xing16’, ‘Dongzao’ × ‘Linyilizao’, ‘Dongzao’ × ‘Jinsi 4’, and ‘Dongzao’ × ‘Yinshanhong’^17,90–92^. The male sterility and kernelless traits are controlled by homozygous recessive genes, and some QTL loci of quantitative traits have also been identified^92–95^.

#### Releasing new cultivars with various maturation times and usages

In the past 30 years, a total of ~200 new cultivars with large fruit, good fruit quality, high resistance to diseases, and varying uses and maturity times were released through polyploid breeding or selection from seedlings, bud mutants, and local germplasms^2^. Among them, the tetraploids ‘Chenguang’, ‘Hongguang’, ‘Riguang’, and ‘Zhuguang’, bred by Hebei Agricultural University, have been awarded new plant variety rights by the National Forestry and Grassland Bureau of China. The fruits of the tetraploids were 30–50% larger in size, 4–7 days earlier to mature and better tasting than diploid fruits.

A large number of local cultivars, such as ‘Zanhuangdazao’, ‘Linyilizao’, ‘Dongzao’, ‘Qiyuexian’, ‘Junzao’, ‘Huizao’, etc. have been excavated and utilized, which has greatly promoted the rapid development of the jujube industry. Recently, some excellent new cultivars for fresh eating, such as ‘Jinsi 4’, ‘Yueguang’, ‘Zaohongmi’, ‘Zaocumi’, and ‘Zaoqiuhong’, and some for dehydration, such as ‘Yuangling 2’, ‘Shuguang’, ‘Zanshuo’, ‘Yushuai’, and ‘Linhuang 1’, with larger fruit, higher quality and higher resistance to fruit diseases than traditional cultivars, have become the dominant cultivars. These dominant cultivars have replaced the traditional cultivars, which has greatly improved the cultivar structure in China.

#### Constructing a high-efficiency propagation system

A set of new propagation approaches has been developed on the basis of traditional sucker division. Among them, stimulating sucker propagation by cutting off the roots at the periphery of the vertical projection of the canopy and separating fasciculate suckers can increase the reproductive coefficient, while gathering and nurturing the suckers in a nursery can greatly improve the quality of the root system. Jujube hardwood cuttings are quite difficult to propagate, presenting a rooting rate of usually <30%, and have rarely been used in commercial production. However, green shoot cuttings (semilignified primary extension shoots, secondary shoots, and bearing shoots) root much more easily and have a high reproductive coefficient. The rooting rate can reach as high as 95%^96^, but its cost and technical requirements are relatively higher than those of other propagation techniques.

Judging from old grafted jujube trees, grafting has been used for at least 1000 years. To meet the needs of large-scale development, grafting propagation with sour jujube as the rootstock has been widely used since the late 1980s. In particular, the use of sour jujube seeds, rather than pits, to obtain rootstocks has become the mainstream method; using this method, the seedlings grow faster and more uniformly than in rootstocks obtained from pits^2^. The use of *Paliurus hemsleyanus* Rehd. as rootstock can also play a role in the prevention of witches’ broom in jujube in southern China^97^. Jujube has also been successfully grafted onto Indian jujube (*Z. mauritiana* Lam.) in subtropical and tropical regions.

The tissue culture of jujube began in 1978. In vitro plantlets were obtained from the stem segments of root suckers in 1983^98^. After 1995, research on jujube tissue culture increased rapidly, and tissue culture with stem tips, stem segments, leaves, anthers, embryos, and cotyledons as explants were all successful^83,86,87,99,100^. However, propagation via tissue culture has not been used on a large scale in jujube in China because of the high technical requirements, high cost, and late fruiting of micropropagated plants.

### Cultivation model and orchard management

Research on jujube cultivation technology has a long history that includes fruitful achievements and has played an important role in promoting the jujube industry. The biological characteristics of jujube are basically understood^1^. To date, cultivation technology systems have been established for the leading cultivars in their main growing areas with their own characteristics. High-density planting and protected cultivation systems have also been applied commercially after the beginning of the 21st century.

#### The unique growth and fruiting habits of jujube

Since the 1950s, the biological characteristics of jujube have been studied comprehensively, and a complete theoretical system had been formed by the 1990s^1,101^. It was revealed that jujube has very strong resistance to abiotic stress, including drought, barren soils, and saline and alkali conditions. It has unique branch and bud characteristics, i.e., usually only the primary shoots can extend, the dormant buds have a very long life, the secondary shoots die back naturally each, the mother-bearing shoots can only extend by ~1 mm per year, and the bearing shoots fall off in the fall. Its flower bud differentiation and fruit set habit are also very distinct, with a short flower bud differentiation time (10 days), a 2-month flowering season, and a low fruit set of only ~1%.

#### Traditional orchard improvement and high-density orchard construction

**Fig. 4:**
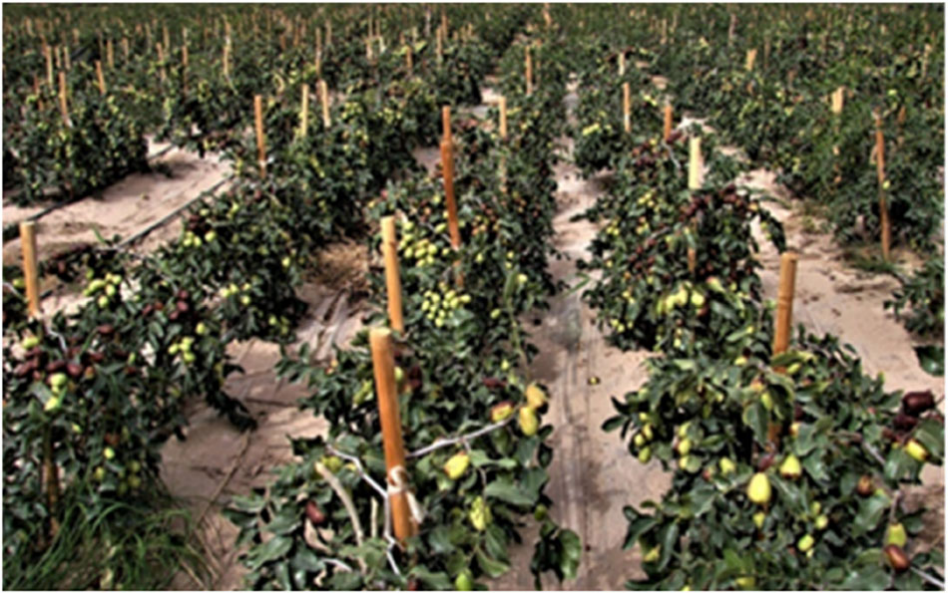
Super high-density jujube orchard in Xinjiang, China (Photo by Prof. Yanjiang Shi)

Since the 1980s, various cultivation techniques focusing on high yield were developed for the leading cultivars and main production areas, which increased production by over 50%, increased the high-quality fruit rate by over 30%, and reduced pesticide use by over 50%^102,103^. At the same time, traditional sparse planting systems with large crowns (row spacing and plant height above 5 m) and intercropping jujubes with cereal crops (row spacing ≥12 m) were replaced by dense dwarf planting (2 m × 3 m) and monoculture orchards. After the Asian Olympic Games in Beijing in 1990, dense cultivation was developed for dwarf fresh jujube, and even superdense plantings (grass orchards) with densities of up to 15,000 plants per hectare were established. After entering the 21st century, in the desert of southern Xinjiang Province, China, a novel cultivation model for high early yields and high fruit quality was established. This method is characterized by the direct sowing of the rootstock seeds (sour jujube) in orchards followed by the in situ grafting of the target cultivar. Starting with a superhigh density (0.5 m × 1.0 m), the density is gradually decreased to 1.0–1.5 m × 4.0 m. This new model obtains good yields (5–8 t/ha) in the year of grafting (Fig. 4) and maintains the high yield at over 15 t/ha 3–5 years later, which is 3–5 years earlier than this yield could be achieved in a traditional orchard^104^.

#### Protected cultivation systems for fresh jujube production

After entering the 21st century, protected cultivation techniques for fresh jujube have developed gradually. Plastic house and Chinese solar greenhouse cultivation (a plastic house with thick back wall as a thermal mass) have been successful in North China and have formed large-scale production regions of over 10,000 ha in Dali County in Shaanxi Province and Linyi County in Shanxi Province in China. In this case, the maturity period can be advanced by 1–4 months^105,106^. These techniques effectively solve the problem of a short supply period for open field cultivation and can increases revenues by 3–5 times. Solar greenhouses along hillsides facing the sun in the Taihang Mountains of Hebei Province have the advantages of lower investment costs, better sunlight, and much better heat retaining properties than a traditional greenhouse by using the mountain as the back wall of the greenhouses^107^. The cultivation of fresh jujubes in plastic shelters has resulted in great success in rainy southern China^108^; this method can greatly reduce the fruit cracking caused by rain at the maturity stage from 70 to <10%.

For fresh eating jujubes, it was found that promoting the lignification of bearing shoots through extremely heavy pruning may accelerate fruiting, increase yield and result in larger fruit^109^. This technique has become a common practice in fresh jujube production. On the other hand, the lignification of bearing shoots changes the deciduous habit of bearing shoots and increases the pruning labor costs.

### Pest and disease management

According to a comprehensive investigation, more than 100 pests and diseases have been observed in jujube^1^. Only approximately ten of them cause severe yield and quality losses, such as peach fruit moth, jujube inchworm, *Ancylis sativa* Liu*, Tetranychus viennensis* Zacher, jujube rust, jujube witches’ broom, and fruit cracking. After the beginning of the 21st century, outbreaks of pests and diseases, including *Lygocoris lucorum, Euzophera batangensis*, *Ceratitis capitata*, jujube flies, fruit cracking, and fruit shrinking, have become increasingly severe^102,110–114^.

High-efficiency management systems for the main diseases and pests in jujube mentioned above have been established in China^3,46,103^. However, high-efficiency, low-cost practical management systems for fruit cracking and fruit shrinking disease have not been developed; these conditions have a great influence on jujube yield and quality. In addition, due to the economic decline of the comparative benefits of jujube, jujube witches’ broom is becoming serious again in orchards due to poor management.

### Postharvest physiology and fresh storage

#### Revealing the postharvest physiological characteristics of jujube fruit

Jujube is difficult to keep fresh. Under normal room temperature and humidity conditions, fresh jujube loses moisture quickly and loses crispness within 3–5 days. It ferments readily and lose firmness when it is tightly sealed in conventional plastic bags under cold storage. A postharvest physiological study revealed the main factors influencing fruit preservation in jujube^115^. At present, there are two opposing viewpoints about the respiratory type of jujube fruit, i.e., climacteric or nonclimacteric, even for the same cultivar (‘Dongzao’)^116–118^.

#### Preservation technology systems for fresh jujube

Many reports on fresh fruit preservation techniques in jujube have been published. The practical techniques include cold storage, controlled atmosphere storage, decompression storage, and controlled freezing-point storage^119–122^. Qu et al. reported that ‘Shanxixiaozao’ could be stored for 70 days at 0 ± 1 °C and 60% RH^119^. Chen et al. indicated that jujube was sensitive to CO_2_ and that fruit browning occurred quickly under the conditions of 10% CO_2_^120^. Zhang et al. found that the respiration rate, ethylene release, and cell membrane permeability of ‘Dongzao’ stored at 0 ± 0.5 °C were significantly inhibited compared with those of jujube stored at room temperature^121^.

Hypobaric storage can delay ripening and aging and inhibit the fermentation of jujube fruit by providing low-temperature, low-oxygen storage conditions^123^. Wang et al. proved that the best storage conditions for fresh jujube were at a temperature of −1 to −2 °C, relative humidity of 95%, 2% O_2_ and 0% CO_2_^124^. Chen et al. showed that the percentage of healthy fruit and the edible rate of ‘Dongzao’ jujube stored at −2 °C for 100 days were 7.4% and 20.2% higher than those of jujube stored at 0 °C^125^. Fu found that controlled freezing-point storage was better than normal cold storage in terms of delaying ripening and aging^126^. Currently, under the optimal storage conditions, half-red fresh jujube can be preserved for 2–3 months or even more than 4 months.

However, in addition to the storage conditions, the duration of fresh jujube storage and the percentage of fruit losses are also influenced by the preharvest cultivation technology and directly by the pathogen load on the fruit in the orchard^14,100,113,127,128^.

#### Postharvest treatments

In the past 20 years, postharvest treatment technology for Chinese jujube has made great progress. Fruit drying technology has gradually changed from traditional natural drying under the sun to dehydration in a drying room or by drying machine^129^. It takes ~1 month for natural drying or air drying to be complete, while the time required can be reduced to 1 day or even less with artificial drying. Compared with those under natural drying, the preservation rates for vitamin C, total sugar, sucrose, fructose, glucose, soluble protein, and other nutrients under artificial drying are significantly improved^130–132^.

In recent years, equipment for jujube fruit sorting or integrated cleaning and sorting has been developed and widely applied in the jujube industry in China^133–135^. The combination of artificial drying, mechanical cleaning, and automatic sorting can significantly improve the appearance of commercial fruit, increase production efficiency and economic benefits, and reduce losses after harvest.

### Nutritional analysis and processing

#### Dominant nutrients and their spatiotemporal distribution

To perform nutritional analysis and intensive processing on jujube, efficient extraction and determination methods were established and optimized for 23 kinds of nutrient components (7 vitamins, 3 triterpenic acids, 8 amino acids, aromatics, cAMP, polysaccharides, flavonoids, and pigments) in jujube^54,136–142^. Converting dehydroascorbic acid to AsA (vitamin C) resolved the bottleneck problem in the determination of dehydroascorbic acid, and accurate determination methods for the two kinds of vitamin C in jujube were finalized^141,143^. The nutrient components in different organs, different fruit developmental stages, and different varieties were systematically analyzed^53,54,144–147^. Jujube leaves are rich in leucine, vitamin B6, carotene, betulinic acid, and ursolic acid, the flowers are rich in vitamin B1 and leucine, and mature fruits are rich in cAMP, functional sugars, vitamin B, triterpenic acid, proline, and some other important functional components in addition to the well-known carbohydrates and vitamin C.

Jujube is an important traditional herb and tonic. Comprehensive studies have shown that the most advantageous nutritional features of jujube fruit include its contents of soluble sugars (2–3 times the levels in other fruits), vitamin C (100 times the level in other fruits), cAMP (1000 times the level in other fruits), vitamin B, triterpenoid acid, proline, polysaccharide, flavonoids, iron, potassium, calcium, and zinc. Therefore, jujube has broad prospects as a material for the development of healthy foods with high nutrition value.

#### Varied processing techniques

There are many traditional processed jujube products, such as candied jujube, smoked jujube, stoneless sugared jujube, jujube liquor, liquor-saturated jujube, jujube jam, jujube paste, and so on^103,148,149^. In the last 30 years, various new products, such as jujube juice, jujube powder, jujube slices, jujube tea, jujube beer, jujube essence, and jujube pigment, have been developed^149^.

After the beginning of the 21st century, much more attention was paid to intensive processing that highlights the characteristic nutrients of jujube. A technique for the sorted extraction and comprehensive wasteless utilization of the main functional components of jujube has been developed on the basis of systematic nutrient composition analysis^54,138,139^. This new technology synchronously produces multiple products such as cyclic nucleotides, dietary fiber, and jujube oil from one raw material^103^. A series of new products with high nutrient value, such as refined jujube cyclic nucleotides, jujube polysaccharides, jujube oil, instant jujube powder, and high vitamin C juice, have been developed^54,139,149^.

## Future research prospects

### Research challenges in the new era

At present, the jujube industry in China has entered a new era of transformation, upgrading, and globalization^150,151^. Given the demands of the new era, the development tendencies of the modern fruit industry and the new technologies available, jujube research in China is facing a series of important challenges. (1) Fundamental research is still weak, and the role of research in supporting major technological innovations is not strong enough. For instance, the formation and genetic mechanisms of the main economic traits of jujube and the interaction between jujube fruit development and environmental changes are still unclear, which restricts breeding efficiency improvements and innovation in cultivation techniques. (2) The modern and highly efficient breeding system is still not fully established, and it is difficult to meet the demands of accelerated cultivar development. (3) Current labor-intensive cultivation techniques were developed with the goal of increasing yields on small farms with inexpensive labor. They rely on increased chemical input and are not environmentally friendly. Moreover, they cannot be adapted to large-scale, mechanized, and labor-saving operations in the future, nor can they meet the demands for high-quality, safe, and high-end products. (4) The low-level processing techniques that imitate those used for other fruits and focus on solving the overproduction problem do not meet the requirements of postharvest value addition and demands for diversified, advanced, and internationalized new products. (5) Macroscopic research on jujube is still quite weak, and the existing studies in nontraditional fields such as ecological and cultural studies and the integration of primary, secondary, and tertiary industries are insufficient. Consequently, it is difficult to fulfill the new demands of government and enterprises for scientific decision-making. (6) International exchange and cooperation are still insufficient and are accompanied by severe imbalances among countries both in production and research. Research is lagging and does not match the demand from industry in some countries. Jujube needs to be promoted to consumers around the world as a ‘new superfruit’ by scientists and commercial companies with the aim of introducing its unique qualities for human health and longevity. The results of sensory tests and analyses with common consumers of different ages and origins are positive and encouraging^14,152^.

### Suggestions for jujube research in the new era

Jujube research in the future should more precisely respond to the needs of industry, markets, and government and could even actively create and lead these demands. Research objectives should be more advanced and diversified. Research content should be more systematic and cover both micro- and macro-issues. Research methods should involve more new approaches, such as modern information technology and multiomics techniques. Research methods should become more synergistic and internationalized. Research results should be more original and advanced. Moreover, fundamental, applied, developmental, and broad-scale visual research should be highly integrated.

The basic ideas for jujube research in the future are as follows: serve the producers, consumers, marketers, government, and society at the same time; learn from experiences with other fruit trees; align with modern global agricultural development, including the reduction of chemical pesticides and environmentally friendly approaches^153^; carry out innovative research in various fields, multiple levels, and through whole industrial chains; and strongly support the advancement of science and technology for the jujube industry in the new era.

In the years to come, jujube research can focus on the following eight aspects:

(1) Multiomics analysis of the formation mechanisms of the important unique traits of jujube to provide a solid foundation for molecular breeding, high-efficiency cultivation, and high-nutrition processing.

(2) High-efficiency breeding technology systems for optimizing and upgrading the cultivar and rootstock structure: integrating the precise identification and utilization of excellent germplasm and the comprehensive application of innovative breeding approaches with rapid evaluation and screening methods; establishing high-efficiency propagation systems for transplant shock-free large nursery plants by integrating the use of containerized seedling production, industrial tissue culture rapid propagation, and micrografting^153^; registering, protecting and promoting newly released varieties worldwide; and including the top varieties in ‘Club’ propagation, production, and commercialization systems, following experience in apple, kiwi fruit, etc.

(3) Novel cultivation technology: developing high-density, high-efficiency, high-quality, labor-saving, and environmentally friendly large-scale, enterprise-oriented operations with the aid of mechanized, automated, and ‘smart’ new technologies.

(4) Year-round fresh fruit supply: integrating the selection of cultivars with different maturation times, half-red harvesting, shelf life preservation, long-distance cold transportation, and long-term storage techniques such as controlled freezing-point storage, as well as protected cultivation, for advancing or delaying ripening and preventing fruit cracking.

(5) Environmentally friendly and high-efficiency pest management integrating physical, agricultural, and biological approaches: minimizing the use of chemical pesticides and reducing chemical residues; promoting labor-saving and low-cost pest and disease control systems; and ensuring high yields, high fruit quality, and food safety.

(6) Quality and nutrition preservation with postharvest and processing technology: adapting to internet and international marketing and upgrading the jujube market structure; making jujube an essential raw material in several ‘superfoods’ that are increasingly requested by the market in addition to being directly used as fresh or dried fruit^13^; extending different production and processing certification systems (e.g., Global GAP, Organic, etc.) for jujube products to respond to the demands of different international markets and especially of large distribution channels.

(7) Nontraditional research fields: integrating the whole industry chain, the new fields of value-added products, related advanced technologies, and technical standards.

(8) International exchange and cooperation: enhancing global jujube research, production, and marketing; promoting more international research, including multicountry, multisite variety, and technology testing, within bilateral agreements and especially with the help of the Jujube Working Group of the International Society for Horticultural Sciences.
